# Quantifying the Robustness of Vegetation Indices through Global Sensitivity Analysis of Homogeneous and Forest Leaf-Canopy Radiative Transfer Models

**DOI:** 10.3390/rs11202418

**Published:** 2019-10-18

**Authors:** Pablo Morcillo-Pallarés, Juan Pablo Rivera-Caicedo, Santiago Belda, Charlotte De Grave, Helena Burriel, Jose Moreno, Jochem Verrelst

**Affiliations:** 1 Image Processing Laboratory (IPL), Parc Científic, Universitat de València, 46980 Paterna, València, Spain; 2 CONACyT-UAN, Secretaría de Investigación y Posgrado, Universidad Autónoma de Nayarit, Ciudad de la Cultura Amado Nervo, Tepic 63155, Nayarit, Mexico

**Keywords:** global sensitivity analysis, vegetation indices, PROSAIL, INFORM, ARTMO

## Abstract

Vegetation indices (VIs) are widely used in optical remote sensing to estimate biophysical variables of vegetated surfaces. With the advent of spectroscopy technology, spectral bands can be combined in numerous ways to extract the desired information. This resulted in a plethora of proposed indices, designed for a diversity of applications and research purposes. However, it is not always clear whether they are sensitive to the variable of interest while at the same time, responding insensitive to confounding factors. Hence, to be able to quantify the robustness of VIs, a systematic evaluation is needed, thereby introducing a widest possible variety of biochemical and structural heterogeneity. Such exercise can be achieved with coupled leaf and canopy radiative transfer models (RTMs), whereby input variables can virtually simulate any vegetation scenario. With the intention of evaluating multiple VIs in an efficient way, this led us to the development of a global sensitivity analysis (GSA) toolbox dedicated to the analysis of VIs on their sensitivity towards RTM input variables. We identified VIs that are designed to be sensitive towards leaf chlorophyll content (LCC), leaf water content (LWC) and leaf area index (LAI) for common sensors of terrestrial Earth observation satellites: Landsat 8, MODIS, Sentinel-2, Sentinel-3 and the upcoming imaging spectrometer mission EnMAP. The coupled RTMs PROSAIL and PROINFORM were used for simulations of homogeneous and forest canopies respectively. GSA total sensitivity results suggest that LCC-sensitive indices respond most robust: for the great majority of scenarios, chlorophyll a + b content (Cab) drives between 75% and 82% of the indices’ variability. LWC-sensitive indices were most affected by confounding variables such as Cab and LAI, although the equivalent water thickness (Cw) can drive between 25% and 50% of the indices’ variability. Conversely, the majority of LAI-sensitive indices are not only sensitive to LAI but rather to a mixture of structural and biochemical variables.

## 1 Introduction

In optical remote sensing, vegetation indices (VIs) are by far the oldest, most studied and largest group of biophysical variable estimation methods using spectral reflectance data [1]. The main reason for their widespread use is their inherent simplicity. The rationale behind the usage of VIs is that these are spectral indicators defined to enhance spectral features sensitive to a vegetation property while reducing undesired effects [2]. A detectable vegetation property can be either a leaf biochemical or a canopy structural variable, such as leaf chlorophyll content (LCC), leaf water content (LWC), or leaf area index (LAI). Nevertheless, the spectral response of a vegetated surface is driven by a complex interplay of absorption and scattering effects [3]. In this respect, indices try to maximize the sensitivity of the variable of interest while minimizing the role of confounding factors. These confounding factors are related to variations of other leaf or canopy properties, background soil reflectance, solar illumination and atmospheric composition (e.g., [4–6]). Although multiple studies have compared the predictive power of VIs for variables of interest (e.g., [7–10]), only few attempted to explicitly quantify the role of confounding factors (e.g., [11–13]).

Apart from relying on VI-based statistical relationships, since the advent of optical remote sensing, efforts have been undertaken to develop physically-based radiative transfer models (RTMs) to understand the propagation of electromagnetic radiation through different media. With respect to the science of vegetation-light interactions, RTMs have been developed at the leaf, canopy and atmosphere scales, and these RTMs can be coupled so that light interactions can be propagated from the leaf throughout the canopy and eventually throughout the atmosphere, e.g., in the direction of a sensor. By coupling a leaf with a canopy RTM, a simulation can serve to facilitate the interpretation of vegetation reflectance in terms of biochemical and biophysical characteristics. For instance, multiple leaf-canopy scenarios can be simulated, thereby varying both leaf biochemical and canopy structural variables [14,15]. Consequently, the usage of RTM simulations proved useful in a wide range of applications, including designing new VIs, performing sensitivity analyses and developing retrieval strategies to infer vegetation properties from remotely sensed data [8,16–19]. Hence, this implies that using RTMs would be the logical choice to analyze the robustness of VIs given varying canopy scenarios, taken into account that models are simplifications of reality. To achieve this in a systematic way, i.e., considering the role of all leaf and canopy variables, an exhaustive sensitivity analysis is required.

A sensitivity analysis can be defined as the process of determining the effect of changing the value of one or more input variables, and observing the effect that this has on the considered model’s output. Sensitivity analysis methods can be categorized as either ‘local’ or ‘global’. Local sensitivity analysis (LSA) methods are often referred to as “one-factor-at-a-time”, because they involve changing one input variable at a time whilst holding all others at their default values, then measuring variation in the outputs. A drawback of LSA methods is that they are informative only at the default point, where the calculation is executed, and do does not encompass the entire input variable space. Thus, LSA methods are inadequate when aiming to quantify the role of all variables considered in the model [20–22]. Unlike LSA, global (variance-based) sensitivity analysis (GSA) explores the full input variable space [20]. Variance-based sensitivity analysis methods aim to quantify the amount of variance that each input variable contributes to the unconditional variance (variance across all simulations) of the model’s output [22]. The approach quantifies the sensitivity to each of the model variables and their interactions. A GSA is thus preferred to identify the driving variables of an RTM and thus to analyze the sensitivity of VIs towards a biophysical descriptor relative to interference factors.

Although earlier GSA studies of RTMs enabled to identify the driving input variables in determining the variability of the spectral outputs (e.g., [23–25]), so far only a few studies translate GSA results into practical remote sensing applications (e.g., [26]). In this work, we aim to use GSA for analyzing the sensitivity of VIs to intended variables and their robustness to confounding factors. Hence, analogous to analyzing the spectral output it could also be applied to analyze the sensitivity of new VIs, and for a specific sensor band setting. A few similar initiatives [27–29] already analyzed the sensitivity of the normalized difference vegetation index (NDVI), which is by far the most widely used index, and a few other common indices to their sensitivity to LAI and LCC, given a specific sensor. However, a systematic analysis of common VIs in view of sensor band settings of operational Earth observers such as the Sentinels, Landsat or MODIS is lacking.

A reason why so far only a few GSA studies were conducted may lie in the absence of a user-friendly toolbox that enables calculating GSA for any VI and any sensor configuration. The lack of a comprehensive VI analysis toolbox was also a motivation to undertake this study and develop a software framework. To do so, we built further on existing GSA codes. As part of the scientific graphical user interface (GUI) toolbox called ARTMO (Automated Radiative Transfer Models Operator [30]), an extension of the already existing GSA toolbox [31], has been developed. In ARTMO, multiple leaf- and canopy-RTMs have been brought together and synchronized within a single scientific GUI toolbox. RTMs can be operated in a semi-automatic fashion for any kind of optical sensor operating in the visible, near-infrared (NIR) and shortwave infrared (SWIR) range (400–2500 nm). The GSA toolbox calculates the relative importance of RTM input variables through first-order and total-order Sobol’ indices, according to the method of [32].

However, until now, the GSA toolbox only enabled analyzing RTM spectral outputs, e.g., reflectance, transmittance, radiance outputs, depending on the analyzed RTM. Although these outputs provide insight into the functioning of RTMs, the toolbox has been of limited use for practical applications such as assessing the sensitivity and robustness of VIs to vegetation variables. Bringing this all together, it boils down to the following main objective: to expand the GSA toolbox to enable calculating GSA of common VIs, given the spectral configuration of prevailing terrestrial Earth observation sensors. This work can be broken down into the following sub-objectives: (1) to develop a GSA toolbox dedicated to the analysis of VIs (GSA-VI); and, (2) to calculate GSA of most common indices sensitive to LCC, LWC, and LAI for common remote sensing sensors.

The remainder of this paper is organized as follows. Section 2 introduces the implemented global sensitivity analysis theory, while Section 3 presents the tested VIs. Section 4 outlines the methodology, i.e., the ARTMO software framework, the RTMs PROSPECT4+SAIL (PROSAIL) and PROSPECT4+INFORM (PROINFORM), followed by an experimental setup. The results’ analysis is presented in Section 5. A discussion on the sensitivity and robustness of commonly used indices is provided in Section 6, and Section 7 concludes the work.

## 2 GSA Theory

Several variance-based GSA methods have been presented in the literature, among others the Fourier Amplitude Sensitivity Test (FAST) by [33], (which uses a periodic sampling approach and a Fourier transformation to obtain the variance of a model output and decompose into partial variances provided by each model parameter), the Sobol’ method [34], and a modified version of the Sobol’ method proposed by [32] (both of them based on variance decomposition). This modification contributed to introducing a simple approximation to identify the Sobol’s sensitivity indices. These indices quantify both the main sensitivity effects (first-order effects: *S_i_*, i.e., the contribution to the variance of the model output by each input variables, it measures the effect of varying each variable) and total sensitivity effects (*S_Ti_*, i.e., the first-order effect plus interactions with other input variables) of input variables. This method has been implemented in the GSA toolbox. A description according to [35] is given below.

Formally, we have a model *y* = *f* (**x**), where *y* is the model output, and **x** = [*x*_1_, *x*_2_, …, *x_k_*]⊤ is the input feature vector. A variance decomposition of *f* (·) as suggested by [34] is:

(1) $$\text{𝕍}(y)=\overset{k}{\underset{i=1}{\sum }}V_{i}+\overset{k}{\underset{i=1}{\sum }}\overset{k}{\underset{j=i+1}{\sum }}V_{ij}\ldots +V_{1,\ldots ,k},$$

where **x** is rescaled to a *k*-dimensional unit hypercube Ω^*k*^, Ω^*k*^ = {**x**|0 ≤ *x_i_* ≤ 1, *i* = 1, …, *k*} ; 𝕍(*y*) is the total unconditional variance; *V_i_* is the partial variance or ‘main effect’ of *x_i_* on *y* and given by the variance of the conditional expectation *V_i_* = 𝕍[𝔼(*y*|*x_i_*)]; *V_ij_* is the joint impact of *x_i_* and *x_j_* on the total variance minus their first-order effects. Here, the first-order sensitivity index *S_i_* and total effect sensitivity index *S_Ti_* are given as [20]:

(2) $$S_{i}=\frac{V_{i}}{\text{𝕍}(y)}=\frac{\text{𝕍}\left[\text{𝔼}\left(y|x_{i}\right)\right]}{\text{𝕍}(y)}$$

(3) $$S_{Ti}=S_{i}+\underset{j\neq i}{\sum }S_{ij}+\ldots =\frac{\text{𝔼}\left[\text{𝕍}\left(y|x_{∼i}\right)\right]}{\text{𝕍}(y)},$$

where *x*_~*i*_ denotes variation in all input variables and *x_i_*, *S_ij_* is the contribution to the total variance by the interactions between variables. Following [32], to compute *S_i_* and *S_Ti_* two independent input variable sampling matrices **P** and **Q** of dimensions *N* × *k* are created, where *N* is the sample size and *k* is the number of input variables. Each row in matrices **P** and **Q** represents a possible value of **x**. The variable ranges in the matrices are scaled between 0 and 1. The Monte Carlo approximations for 𝕍(*y*), *S_i_* and *S_Ti_* are defined as follows [22,32]:

(4) $$\hat{\text{𝕍}}(y)=\frac{1}{N}\overset{N}{\underset{j=1}{\sum }}{\left(f{\left(\text{P}\right)}_{j}\right)}^{2}-{\hat{f}}_{0}^{2},\;\;\;{\hat{f}}_{0}=\frac{1}{N}\overset{N}{\underset{j=1}{\sum }}f{\left(\text{P}\right)}_{j},$$

and

(5) $${\hat{S}}_{i}=\frac{1}{N}\sum_{j=1}^{N}\frac{f{\left(\text{Q}\right)}_{j}(f{\left({\text{P}}_{\text{Q}}^{\left(i\right)}\right)}_{j}-f{\left(\text{P}\right)}_{j})}{\hat{𝕍}(Y)},\;\hat{S_{Ti}}=\frac{1}{2N}\sum_{j=1}^{N}\frac{{\left(f{\left(\text{P}\right)}_{j}-f{\left({\text{P}}_{\text{Q}}^{\left(i\right)}\right)}_{j}\right)}^{2}}{\hat{𝕍}(y)},$$

where $\hat{\ldots }$ is the estimate; ${\hat{f}}_{0}$ is the estimated value of the model’s output; defining *f* (**P**) as all outputs for row vectors in **P**; ${\text{P}}_{\text{Q}}^{\left(i\right)}$ represents all columns from **P** except the *i*th column which is from **Q**, using a radial sampling scheme [36]. Matrices are generated with a Sobol distribution [37,38] of size *N* × 2*k* where **P** and **Q** are the left and right half of this matrix, respectively [32]. In order to compute *S_i_* and *S_Ti_* simultaneously, a scheme suggested by [39] was used which reduced the model runs to *N*(*k* + 2).

Both PROSAIL and PROINFORM models generate bidirectional top-of-canopy (TOC) reflectance in the 400–2500 nm, PROSAIL with a spectral resolution of 1 nm as output, i.e., 2101 spectral bands and PROINFORM with a spectral resolution of 5 nm as output, i.e., 421 spectral bands. Based on these data, VIs are calculated and the GSA is run. Each VI is calculated as a new RTM output, whereby for each simulation the reflectance data associated with the bands as defined by the VI formulation are extracted and then the VI calculated. By executing the process according to sensor band settings, additionally, a spectral filter of each one of the bands for each sensor is applied. This improves the accuracy of the sensor-specific VI calculations, but it is at the expense of an intensive processing time.

## 3 Common Vegetation Indices Applied to Operational Sensors

For the last four decades a plethora of remotely-sensed VIs have been published (see [5] for review). Particularly since the advent of remote sensing spectroradiometer data, from which virtually an unlimited number of VIs can be designed, an ever growing variety of VIs have been proposed. While ideally each proposed VI must be analyzed on its sensitivity to a targeted variable relative to preserving robustness to confounding factors, here we will restrict to common VIs that are widely used in remote sensing mapping applications. This implies we restrict the analysis to VIs that are applicable to freely available remote sensing imagery data sources, being optical sensors of contemporary operational multi-spectral Earth-observing satellites. These missions include Landsat 8, MODIS, Sentinel-2 (S2) and Sentinel-3 (S3). The characteristics of these sensors are provided in Table 1. Consequently, only VIs will be analyzed that by their design can be obtained from the band settings of these sensors. Thereby, while the majority of indices can be calculated for all sensors, some can only be calculated from one or two sensors, e.g., S2 or S3. Furthermore, for reasons for brevity, only VIs will be analyzed that claim sensitivity towards LCC, LWC, and LAI. Indices are selected according to the online database https://www.indexdatabase.de created by [40], where indices can be sorted according to variable sensitivity and sensor band settings.

According to these criteria, the following VIs will be analyzed, organized per variable and sensor type: see Table 2 for LCC-sensitive VIs, Table 3 for LWC-sensitive VIs and Table 4 for LAI-sensitive VIs. However, this category is non-exclusive: many more VIs are commonly used, for instance those that merely aim to assess the “greenness” of vegetation rather than claiming sensitive to a specific quantitative variable. Hence, the here followed categorical organization is only indicative.

Considerably more indices can be calculated when moving from broadband sensors towards imaging spectroscopy missions. To illustrate this, indices were selected, Table 5, that can be calculated with the forthcoming Environmental Mapping and Analysis Program (EnMAP) hyperspectral satellite mission [66]. Characteristics are available in Table 1. Despite the number of missions currently under development, the choice of the EnMAP sensor is due to the large amount of information available from a wide variety of articles [67–73], in addition to all the possibilities offered by its large number of bands. Although this mission has not been launched yet, it is of interest to analyze the sensitivity of EnMAP-suited VIs in preparation of future vegetation monitoring applications.

## 4 Methodology

### 4.1 ARTMO’s Software Framework

The entire GSA-VI software development and the conducted analysis were undertaken within the in-house developed ARTMO framework [30]. ARTMO is developed in MATLAB [84] and consists of a suite of leaf and canopy RTMs, retrieval toolboxes and post-processing toolboxes, among which is the GSA toolbox [31]. With this toolbox, any of the integrated RTMs in ARTMO can be analyzed on input-output relationships. Essentially, the GSA toolbox calculates the relative importance of RTM input variables through first-order and total-order Sobol’ sensitivity indices. An essential part of GSA methods is that the RTM parameter space has to be sampled. In the toolbox various sampling distribution methods have been implemented, including: uniform, extreme value, exponential, normal, Latin hypercube sampling and the default Sobol sampling [37,38]. The sensitivity analysis can be employed along the spectral domain for any kind of optical sensor setting within the 400–2400 nm range. In this version (v.1.09), the GSA toolbox has been expanded with a GUI module to analyze vegetation indices. In this module, the user can define any index formulation and, if a sensor is selected, assign the spectral bands to an index. Multiple indices can be as such defined and analyzed at once. Further, a visualization tool has been subsequently developed that enables visualizing the sensitivities of the analyzed indices to the different RTM input variables. Among the RTMs implemented into ARTMO, the PROSAIL model is the most commonly used coupled leaf-canopy RTM [14,15]. Therefore, this model was chosen as baseline model to analyze the selected VIs. PROSAIL is commonly applied to describe the reflectance characteristics of a uniform canopy derived from the combination of PROSPECT-4 [85] leaf model and the SAIL canopy structure model [86]. To account for more heterogeneous canopies, also the INvertible FOrest Reflectance Model (INFORM) [87,88] model was chosen because of its suitability in simulating forest canopy reflectance while preserving a relative simplicity. The models are briefly explained below.

### 4.2 PROSAIL and PROINFORM

PROSPECT-4 calculates leaf reflectance and transmittance as a function of its biochemistry and anatomical structure. It consists of four parameters, those being leaf structure (N), chlorophyll a+b content (Cab), equivalent water thickness (Cw) and dry matter content (Cm). PROSPECT-4 simulates directional reflectance and transmittance over the spectral range going from 400 to 2500 nm at the fine spectral resolution of 1 nm. These outputs serves as input into the SAIL canopy model. SAIL is easy to use due to its low number of input variables. The model is based on a four-stream approximation of the radiative transfer (RT) equations, in which case one distinguishes two direct fluxes (incident solar flux and radiance in the viewing direction) and two diffuse fluxes (upward and downward hemispherical flux) [89]. SAIL inputs consist of leaf area index (LAI), leaf angle distribution (LAD), ratio of diffuse and direct radiation, soil coefficient, hot spot and sun-target-sensor geometry, i.e., solar and observer zenith angle and relative azimuth angle (SZA, OZA and RAA, respectively). Given that canopy structure is only determined by LAI and LAD, this model is therefore used to simulate homogeneous canopies, e.g., monoculture crop fields. According to a systematic review on the use of PROSAIL for simulating common crops (maize, wheat, rice, soybean, sugar beet) by [15], we constrained the dynamic ranges of the PROSAIL variables, which are wide enough to be representative and realistic for regional agricultural applications, as described in Table 6.

Regarding the simulation of forest canopies, INFORM was also coupled to PROSPECT-4. INFORM is a hybrid model combining the strengths of the turbid-medium and the geometric-optical radiative transfer models. It couples the SAILH [86] model which simulates the radiative transfer within the turbid-medium canopy layer with the FLIM [90] model to account for geometric aspects such as leaf clumping inside, tree crowns and crown geometry. When coupled with PROSPECT-4 (PROINFORM), the model simulates the forest as a function of the aforementioned leaf-level variables, as well as the canopy-level variables, i.e., LAI of the single trees (LAIs), LAI of the understory (LAIu), average leaf angle (LAD), tree height (H), crown diameter (CD), stem density (SD), besides other parameters describing the sun-sensor geometries and irradiance conditions, i.e., sun zenith angle (SZA), observer zenith angle (OZA), relative azimuth angle (RAA) and fraction of diffuse radiation.

### 4.3 Experimental Setup

After selecting the indices and targeted variables defined, the GSA settings need to be defined. As mentioned above, the variance-based GSA method of [32] was implemented. The PROSAIL variable boundaries from Table 6 were inserted with the Sobol sampling scheme, being the standard sampling distribution for calculating a GSA [37]. Finally, the number of samples per variable needed to be defined, which is a trade-off between accuracy and processing time. To identify this trade-off, an initial study was conducted by gradually increasing the sample size. As such, the number of samples when results stabilize can be defined, and set for subsequent VI analysis. Only total effect sensitivity results are shown, *S_Ti_*, thus taking interactions between variables into account.

## 5 Results

### 5.1 Impact of Number of Samples per RTM Variable on GSA

**Figure 1:**
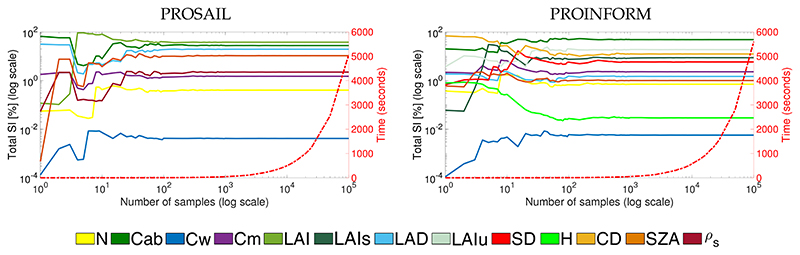
Analysis of the impact of number of samples on global sensitivity analysis (GSA) stability. GSA has been run for NDVI with PROSAIL (**left**) and PROINFORM (**right**), increasing the number of samples.

Because the complexity of a model and the number of simulations exert influence on the GSA results, it is important in GSA studies to identify where sensitivity results stabilize. To do so, a GSA was run with NDVI calculated from PROSAIL and PROINFORM simulations whereby the number of samples has been gradually increased. GSA results (*S_Ti_*) are shown in Figure 1 in log scale for both axes. It can be noted that around 1500 samples all variables stabilize. Specifically, no more fluctuations occurs after 2000 simulations per variable. Hence, all subsequent analyzes were carried out with 2000 samples per variable (according to [39] adds up to a total of 20,000 simulations for PROSAIL and 28,000 for PROINFORM, Section 2). Although this is a rigorous approach, since the models run fast the GSA processing time was reasonable (about 2 min), and we can be sure that no biases due to the method instability have been introduced. The Y-axis (*S_Ti_*) is plotted in log-scale because the majority of variables appear to be of negligible importance. In fact, PROSAIL-based NDVI is predominantly driven by two variables: Cab and LAI. These two variables alone determine over 65% of the NDVI variability. This trend is confirmed using PROINFORM, with NDVI being predominantly driven by Cab, LAIs, LAIu and CD; These four variables determine over 80% of the NDVI variability.

### 5.2 GSA S_Ti_ Results along the 400–2500 nm Spectral Range

**Figure 2:**
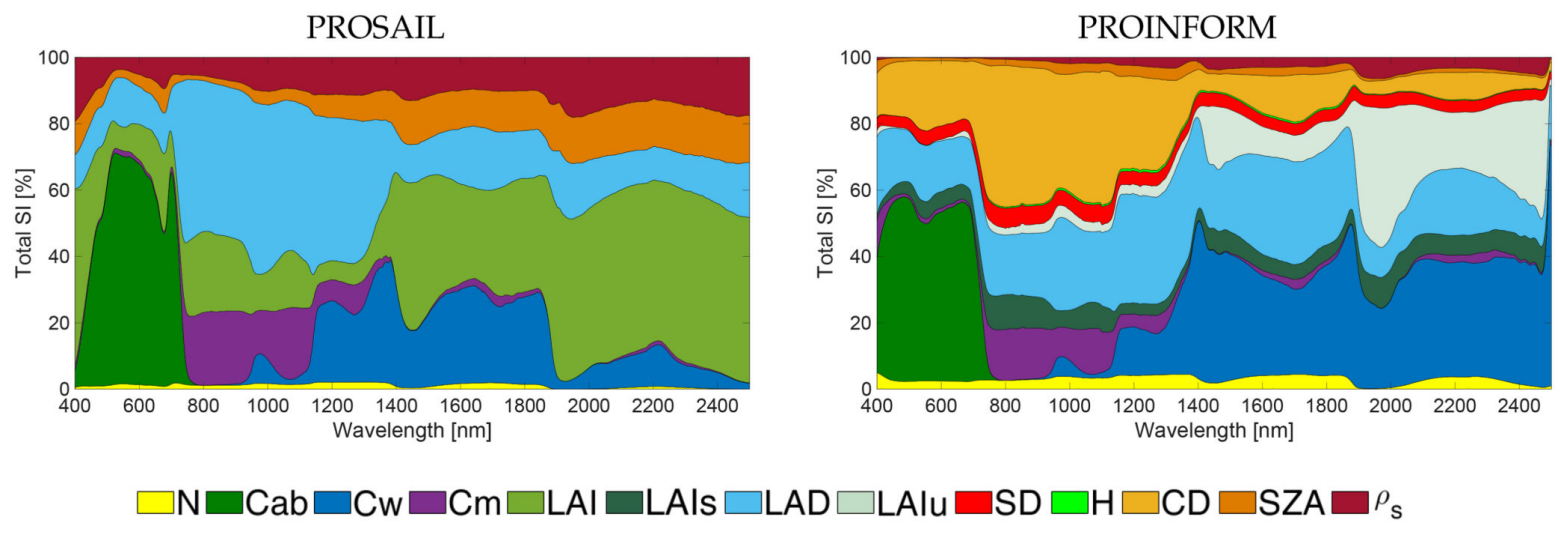
PROSAIL (**left**) and PROINFORM (**right**) *S_Ti_* results along the 400–2500 nm spectral range.

To gain insight in the variables driving RTM reflectance output, first *S_Ti_* results are presented along the spectral range for PROSAIL and PROINFORM (Figure 2). These results not only identify the driving variables along the spectral range, but also identifies the differences in performances between the homogeneous canopy configurations using SAIL and the forest canopy configurations using INFORM. While the leaf model contributions of the canopy scenarios are alike, with strong influence of Cab in the visible, and of Cw in the NIR and SWIR parts of the spectrum, large differences regarding the variables characterizing the canopy structure can be observed. Whereas in SAIL canopy structure is driven by LAI and LAD, for INFORM these two variables play only small role in representing canopy structure. The key structural drivers are crown diameter (CD) and LAI of understory (LAIu). These results can be interpreted as follows. Canopy structure is defined by two layers in INFORM: The first layer represents LAI as a single tree LAI (LAIs). Canopy leaf density is consequently calculated as the product of LAIs, CD, SD and H, having the greatest impact in this process the variable CD [88]. The second layer is defined by the LAI of the understory (LAIu), that fixes the proportion of soil reflectance into the TOC reflectance. In this respect, LAIs and LAD of the canopy play a less important role, as these variables are no longer the key drivers that determine the proportion of vegetation and soil reflectance in the TOC reflectance. Having the overall mechanisms identified, it allows us interpreting the GSA *S_Ti_* results of VIs that are designed to be sensitive to LCC, LWC and LAI.

### 5.3 GSA S_Ti_ Results for LCC-Sensitive Indices

**Figure 3:**
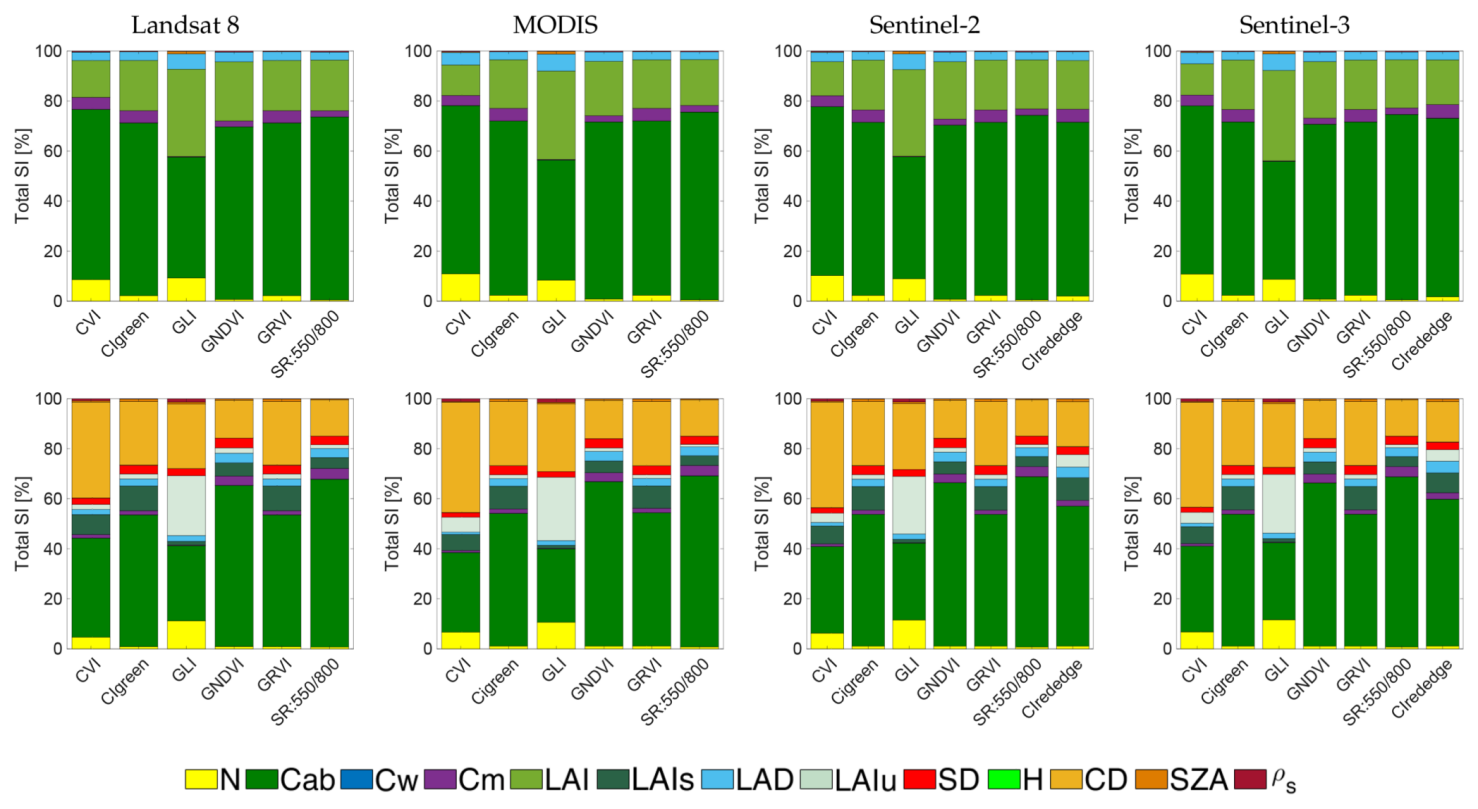
Comparison of leaf chlorophyll content (LCC)-sensitive indices for PROSAIL (**top**) and INFORM (**bottom**) simulations for band settings of four sensors.

Starting the GSA with LCC-sensitive indices, Figure 3 shows the *S_Ti_* results sorted per sensor for PROSAIL and PROINFORM. When inspecting these figures, the following general trends can be observed: for all LCC-sensitive indices, they respond effectively most sensitive to the RTM variable chlorophyll a+b content (Cab) given all ranging RTM variables. Total sensitivities (*S_Ti_*) of Cab are more dominant for PROSAIL than for PROINFORM. This is not surprising given the more structural variables introduced into the forest RTM INFORM, particularly CD plays an important confounding role. For PROSAIL the structural variable LAI is secondly driving the sensitivity of the indices.

Most of the LCC-sensitive indices responded consistently across the tested sensors. For the PROSAIL scenarios, top sensitive indices were the following (with each *S_Ti_*): CIrededge (for S2 and S3 only) (71% for S3), CVI (68% for L8), CIgreen (69% for MODIS), GNDVI (70% for MODIS), GRVI (69% for MODIS) and spatial resolution (SR):550/800 (74% for MODIS), which indicates these indices are highly sensitive to Cab. For the PROINFORM scenarios, however, CVI no longer reacheed a dominance towards Cab, with a value of 34% in the best case. Here, CIrededge (58% for S3), CIgreen (52% for MODIS), GNDVI (65% for MODIS), GRVI (53% for MODIS) and SR:550/800 (68% for MODIS) reached a *S_Ti_* above 50%, with GNDVI and SR:550/800 being the most sensitive LCC indices for both RTMs.

For both PROSAIL and PROINFORM scenarios, the index GLI showed the least sensitivity towards Cab (48% for PROSAIL and 30% for PROINFORM). These models were strongly influenced by structural variables such as LAI in PROSAIL or CD in the case of PROINFORM. When comparing the indices across the four sensors, then only subtle differences can be noticed. Although each sensor was configured with their own band settings in terms of band centre and band width, these differences tend to be of negligible influence in the sensitivity performances of the indices. Thereby, despite that sensors with more bands in the Cab-sensitive region (see Table 1) allow to calculate more LCC-sensitive indices, i.e., CIrededge for S2 and S3, yet this index yielded about the same sensitivities.

Overall, it can be observed that the PROINFORM structural variables suppressed the sensitivity towards Cab. While in PROSAIL, mainly LAI and, to a smaller extent, LAD played a role, in case of PROINFORM CD is the dominant structural driver influencing the response of the indices. In fact, LAI of the understory (LAIu) mostly drove the index for GLI. Summarizing, the inter-comparison analysis suggests that the large majority of LCC-sensitive indices were effectively sensitive to Cab with only marginally affected by structural variables. The indices SR:550/800 and GNDVI responded the most robust, considering the tested sensor settings and the two contrasting canopy scenarios.

### 5.4 GSA S_Ti_ Results for LWC-Sensitive Indices

**Figure 4:**
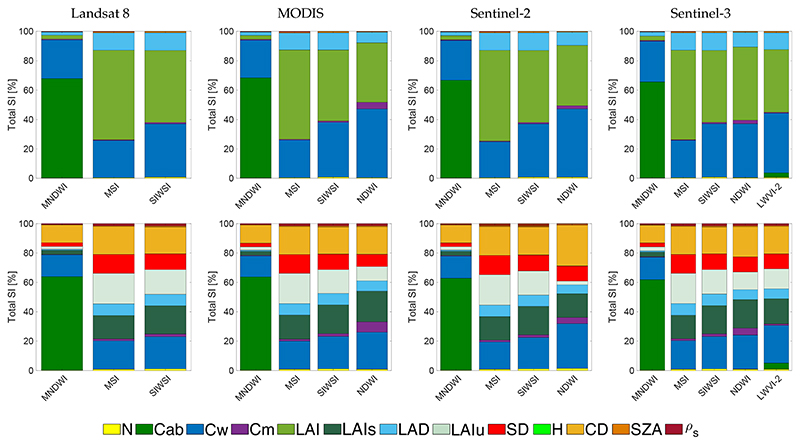
Comparision of leaf water content (LWC)-sensitive indices for PROSAIL (**top**) and PROINFORM (**bottom**) simulations for band settings of four sensors.

Regarding the LWC-sensitive indices, a first observation was that *S_Ti_* sensitivity results show more modest sensitivities towards leaf Cw (Figure 4). While for PROSAIL, all tested indices show sensitivity towards Cw, the relative importance was generally less than 50%. In fact, the majority of these indices responded more sensitive to LAI or Cab than to Cw. Hence, confounding factors are overruling the sensitivity towards Cw. The situation was even worse for the forest scenarios as simulated by PROINFORM.

With PROINFORM forest canopy simulations, despite their overall low sensitivity, the majority of LWC-sensitive indices responded consistently across the tested sensors. This suggests that the role of the sensors was marginal for the tested indices. For the PROSAIL scenarios, top sensitive indices are the following: NDWI and SWSI. Total SI sensitivity can go up to 37%, in case of NDWI with MODIS, only surpassed by LWVI-2 in case of S3, with a *S_Ti_* of 40%. Regarding the PROINFORM scenarios, sensitivities are systematically lower given the more structural variables involved. Only the NDWI index reached a *S_Ti_* above 23% for S2 and over 25% in the case of LWVI-2 with the sensor S3. For this VI, the sensor used is S3-SLSTR, which is equipped with SWIR bands. For both considered RTMs, MNDWI responded the least sensitive towards Cw. Similarly as before, differences can hardly be noticed when comparing the common indices across the four sensors.

Overall, when comparing PROSAIL against PROINFORM, it can be noted that the PROINFORM structural variables suppress the sensitivity towards Cw. In PROSAIL, LAI is the most important structural driver, and often more sensitive than Cw. In the case of PROINFORM, various structural drivers play a role, with CD and then LAIs, LAIu, and SD as the most important variables. Summarizing, the inter-comparison analysis suggests that the large majority of LWC-sensitive indices are somewhat sensitive to Cw, with the index NDWI being the most robust considering the tested sensor settings and the two contrasting canopy scenarios. Yet, structural variables play an even more dominant role in the response of these indices. Hence, this means that in the case of structurally heterogeneous canopies utmost care is required when using and interpreting these indices.

### 5.5 GSA S_Ti_ Results LAI-Sensitive Indices

**Figure 5:**
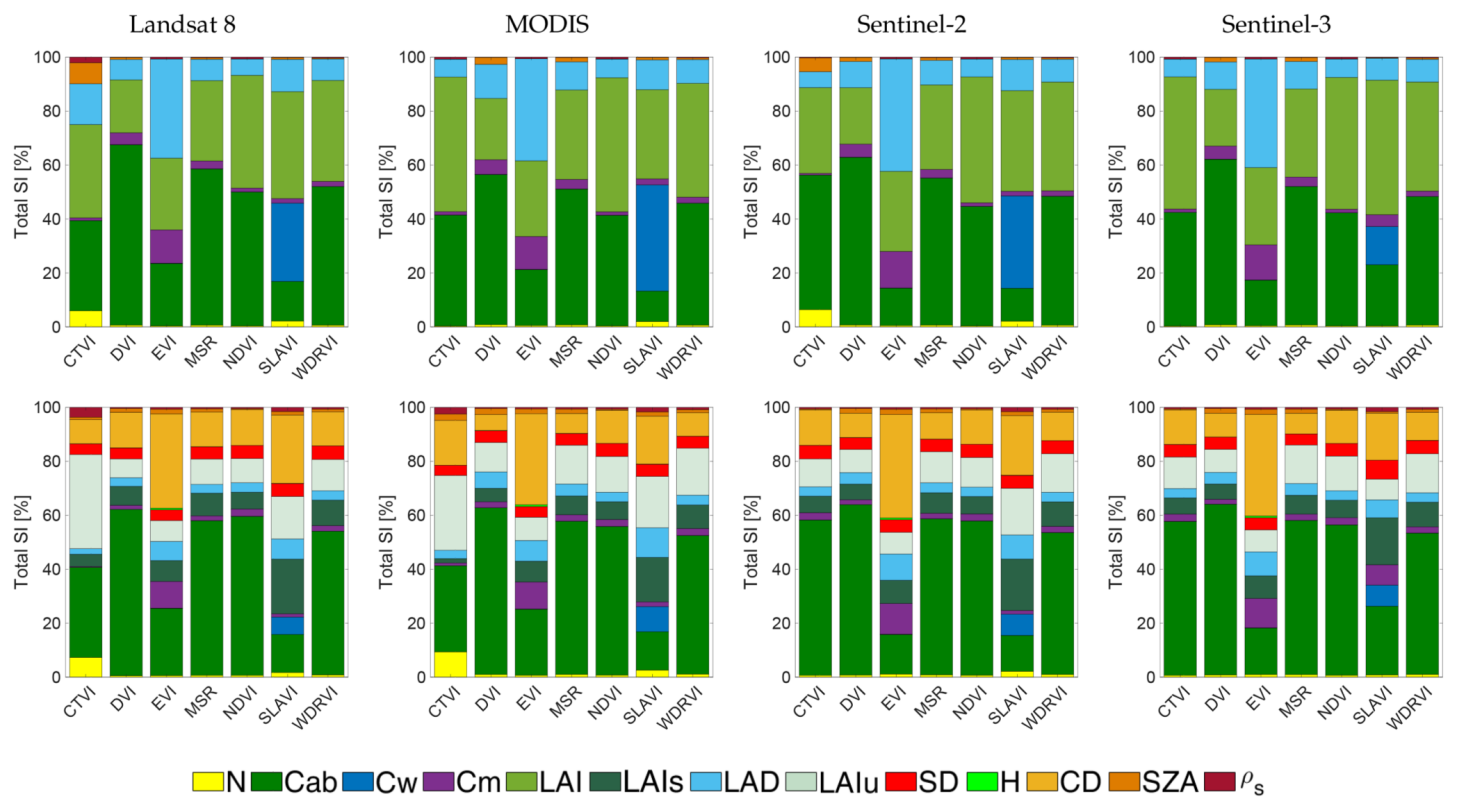
Comparison of leaf area index (LAI)-sensitive indices for PROSAIL (**top**) and INFORM (**bottom**) simulations for band settings of four sensors.

Regarding the LAI-sensitive indices, Figure 5 shows again the GSA *S_Ti_* results sorted per sensor for canopy configurations using PROSAIL and PROINFORM. When inspecting these figures, the following general trends can be observed. In the case of homogeneous canopies, as simulated by PROSAIL, *S_Ti_* results suggest that effectively all analyzed LAI-sensitive indices show a strong sensitivity towards LAI. Yet, results also suggest that for the majority of indices, LAI is not evaluated as the most dominant variable. In fact the LAI-sensitive indices expose stronger sensitivity towards Cab. In the case of forested canopies as simulated by PROINFORM, canopy LAI within a tree (LAIs) shows only marginal influence, it is LAI of the understory (LAIu) that is more driving, given its role of covering soil reflectance. However, canopy structure is in PROINFORM defined by 4 structural variables (LAIs, SD, H, CD) as it was exposed in Section 5.2. These variables together add up to *S_Ti_* values above 50% for all indices, justifying the sensitivity of these indices towards canopy structure.

The majority of LAI-sensitive indices behaved consistently across the tested sensors. For the PROSAIL scenarios, the top sensitive indices are the following with the associated value of *S_Ti_*: CTVI for MODIS with 50% and S3 with 49%, SLAVI for S3 with 49% and L8 with 39%, WDRVI for MODIS with 42%, S2 with 40% and S3 with 40% and NDVI showed high sensitivity among all sensors, being the higher MODIS with a 50%. For these indices, *S_Ti_* can go up to 40%, which indicates these indices were highly sensitive to LAI. The S3 sensor showed great stability, reaching a value of 49% for NDVI, CTVI, and SLAVI, we see this same value repeated in MODIS for CTVI and NDVI. For the PROINFORM scenarios, considering that canopy leaf density is defined by a combination of multiple structural variables (LAIs, SD, H, CD), then SLAVI, CTVI, and EVI are mostly sensitive to canopy structure. CTVI shows a *S_Ti_* of 21% for MODIS, 24% for S2 and 20% for S3, reducing its value to 17% in L8. Also noteworthy is the impact of LAIu, which reaches a *S_Ti_* of 35% for L8 and 28% for MODIS, being higher than the actual contribution of the combined canopy structural variables. EVI responded more stable across all the sensors, with a maximum value of 52% for S2 and a minimum of 46% for MODIS. SLAVI, as well as EVI, showed a robust value across all the sensors (50% for L8, 45% for S2), but also influenced by LAIu (8% for L8 and 9% S2).

For both PROSAIL and PROINFORM canopy scenarios, the DVI and MSR responded the least sensitive towards LAI. Instead, these indices showed a sensitivity towards Cab. Also, WDRVI indices exposed more sensitivity towards Cab than LAI, in the case of PROSAIL this difference is very small, between 5% and 10% depending on the sensor. However, in PROINFORM this difference becomes much more evident, keeping Cab with a value higher than 45% for all the VI and the weight of LAI distributed among all the variables of structure not being superior to 40% of *S_Ti_*. When comparing the indices across the four sensors, overall the sensors band settings lead to the same results. Yet, in case of PROINFORM the CTVI index responded more sensitive to structure for the Landsat-8 and MODIS sensors than for the S2 and S3 sensors (in the case of SLAVI, making use of S3—SLSTR).

Overall, it can be noted that the multiple structural canopy variables as defined by PROINFORM are mostly driving the variability of the LAI-sensitive indices. While LAI was the most important structural driver in PROSAIL, in case of PROINFORM, LAI of the understory (LAIu) and CD acted as the most important variables. Tree height (H) had no impact on any of the indices. Summarizing, the large majority of LAI-sensitive indices were effectively strongly sensitive towards canopy structure, i.e., LAI or other variables that define the structure in case of PROINFORM, with the index SLAVI as the most robust considering the tested sensor settings and the two contrasting canopy scenarios.

### 5.6 GSA S_Ti_ Results for Hyperspectral Indices

**Figure 6:**
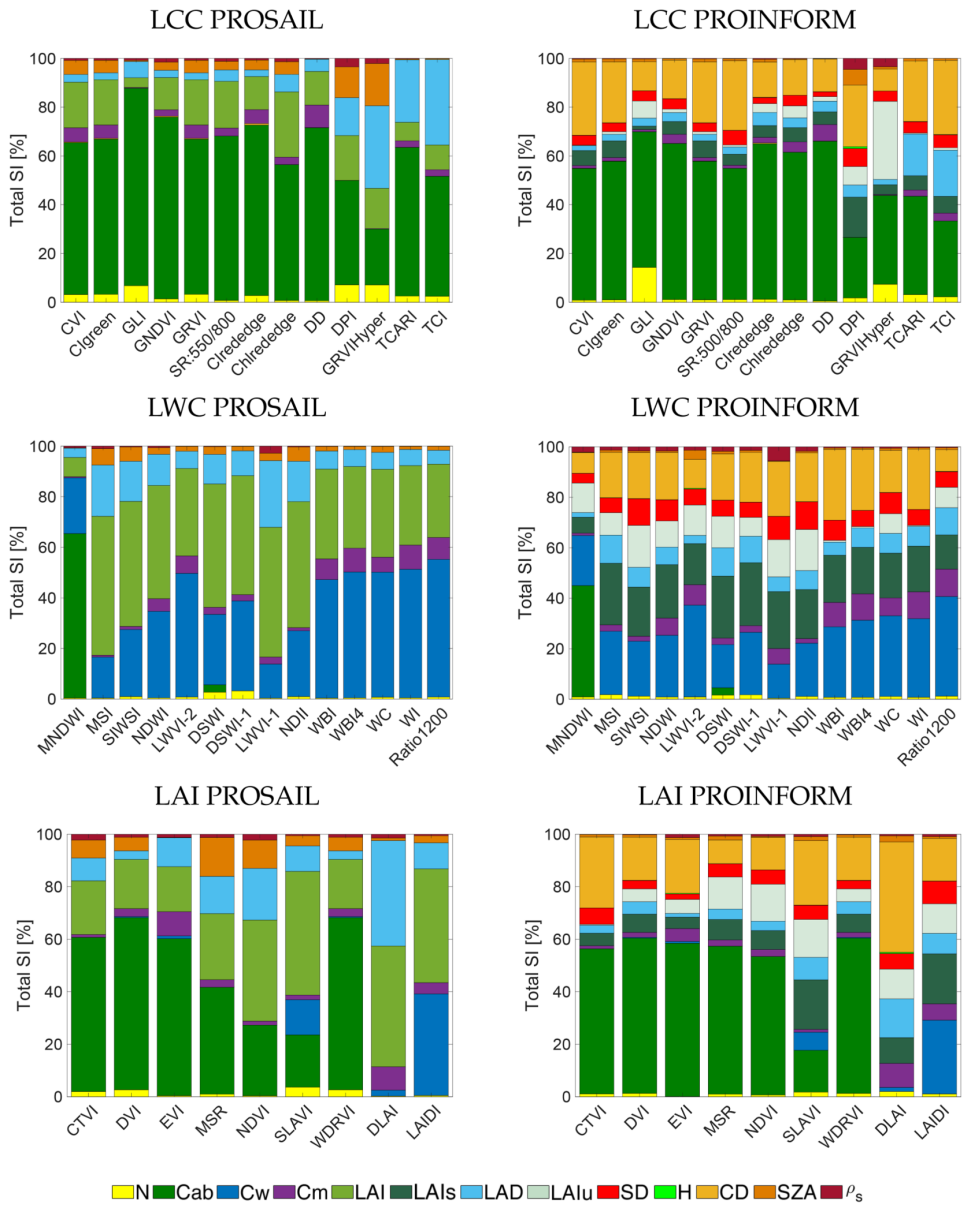
Comparison of GSA results (*S_Ti_*) for LCC (**top**), LWC (**middle**) and LAI (**bottom**) sensitive indices for PROSAIL (**left**) and PROINFORM (**right**) canopy configurations for the EnMAP band settings.

Since the above broadband sensors are configured with a limited number of bands, the number of valid indices that can be derived are limited. Also, because of the overlap in the spectral bands, only small differences were encountered across the tested sensors. Hence, in this section indices are evaluated according to the band settings of the forthcoming EnMAP imaging spectrometer that is configured with 230 narrow bands. Consequently, given the many bands at disposal, not only can more indices sensitive to the variables of interest be calculated, but the probability to evaluate robust indices may also be higher. Results for both PROSAIL and PROINFORM canopy configurations are provided in Figure 6.

An inspection of the LCC-sensitive indices reveals the same excellent performances as observed before. Although no systematic superior performances, as opposed to the broadband indices, emerged, yet some indices showed more sensitivity towards structural variables than to Cab, namely GLI with a *S_Ti_* of 80% for PROSAIL and 55% for PROINFORM. The other tested indices did not reveal a clear advantage, except for the case of Chlrededge (56% for PROSAIL and 60% for PROINFORM) and DD (71% for PROSAIL and 65% for PROINFORM), with a similar result as the one exposed by CIrededge with the S3 sensors.

Many more narrowband than broadband LWC-sensitive indices were found in literature, but only a few of them showed a dominant sensitivity towards Cw. Particularly the following indices exposed a strong sensitivity in the case of PROSAIL (with *S_Ti_*): WBI (47%), WBI4 (49%), WC (49%), WI (50%) and Ratio1200 (54%). This trend can be confirmed in the case of PROINFORM but with lower values; the combined structural variables govern the indices’ variability. Ratio1200 reached the highest sensitivity with Cw *S_Ti_* up to 39%, while among the narrowband indices WC yielded the highest sensitivity with a *S_Ti_* of 32%. It is also worth mentioning that LWVI-2 with *S_Ti_* of 49% for PROSAIL and 36% for PROINFORM responded stronger sensitive towards Cw than the other narrowband indices due to the contribution of a SWIR band. Moreover, in fact, the strongest sensitivities emerged towards structural variables, which are dominating for the majority of indices. This again suggests that although LWC-sensitive indices can estimate Cw, they are also sensitive towards canopy structural heterogeneity.

Regarding LAI, similar as for the broadband indices, a distinction between PROSAIL and PROINFORM has to be made. The following trends were observed: first, compared to the above leaf variables indices, fewer LAI-sensitive indices were found in literature. Second, for the majority of indices, LAI is not the dominant driving variable but rather Cab. Third, for PROSAIL only the indices (with *S_Ti_*) DLAI (46%), LAIDI (43%), NDVI (38%) and SLAVI (47%) emerged to be dominantly sensitive towards LAI. About the same trend is observed with PROINFORM when merging all its structural variables. For PROINFORM, combining the canopy density variables (LAIs, SD, H, CD), DLAI shows the higher total sensitivity towards LAI, with 58% followed by SLAVI with 48%, being the most dominant variables CD and LAIs followed by LAIu with 11% for DLAI and 14% for SLAVI. In fact, noteworthy is that when comparing against the LWC-sensitive indices, these indices show generally a higher sensitivity towards LAI than the here tested LAI-sensitive indices.

## 6 Discussion

After having developed the GSA software framework, we conducted a sensitivity analysis of common VIs that were designed to respond sensitively towards the LCC, LWC, and LAI, respectively. Their robustness against confounding factors was analyzed by running a GSA using PROSAIL and PROINFORM simulations, i.e., representing respectively homogeneous and forest canopy scenarios. Based on the derived results given band settings of common broadband sensors and the selected imaging spectrometer, the following general trends can be observed:

- Regarding LCC-sensitive indices, overall the most robust indices are GNDVI and SR:550/800. Those indices showed the highest total sensitivity to Cab and are thus most robust to the confounding effects of other RTMs variables. Moreover, LCC-sensitive indices are applicable to all the sensors tested including the imaging spectrometer EnMAP. For EnMAP, GNDVI showed an increase of *S_Ti_* up to 74%, as well as GLI, up to 79%. In a related study by [91], GNDVI revealed a similarly high sensitivity towards Cab as well as to LAI, but also small differences can be appreciated between both studies, probably due another GSA method used, named EFAST. When interpreting the results from a sensor point of view, then the broadband indices tend to respond more robust towards Cab estimation than the spectrometer narrowband specific indices. Hardly differences were encountered across the four tested broadband sensors. Yet, a trend can be observed, namely that these robust indices are based on exploiting the bands between 450 nm and 800 nm. This spectral range is where all the processes related to Cab absorption occur [92,93]. Most of these indices make use of only 2 bands: one sensitive band is used in the red or green region and this is compared against a more stable reference band, which is located in the NIR region [74].
- Regarding LWC-sensitive indices, overall the most robust indices are WI and Ratio1200 for PROSAIL, being the only ones that surpass 50% of *S_Ti_* and Ratio1200 for PROINFORM. These are narrowband indices available with EnMAP. Hence, for LWC-sensitive narrowband indices proved to be more effective than broadband indices. The Ratio1200 uses 3 bands located around the 1200 nm water absorption region. The influence of the SWIR band is also observed in the study by [94], as expressed by a high sensitivity of the LWVI-2 index with Cw. It is noteworthy that these indices always use a band in the NIR and SWIR regions, which is related to water absorption [95,96]. A drawback of SWIR-based indices, however, is that only a limited number of sensors cover the SWIR range. Results also suggest that multiple-band indices can be more effective than traditional 2-band indices. For instance, Ratio1200 exploits this relation using the bands: 1205 nm, 1095 nm and 1275 nm. Another remark is that the majority of the LWC-sensitive indices show superior sensitivity towards LAI, even more than some LAI-sensitive indices. This suggests that homogeneous canopies are required for the mapping of LWC [97]. The only index where we observed a good sensitivity across traditional and narrowband indices is NDWI, and also LWVI-2, which is only available for S3 when making use of SLSTR bands.
- Regarding LAI-sensitive indices, overall the most robust index is SLAVI. This index showed the highest overall sensitivity to LAI given the other PROSAIL variables and is applicable to all sensors. The narrowband spectrometer EnMAP dataset yielded somewhat better results than the broadband sensors, with the indices DLAI and LAIDI as best performing. However, when the structure is defined by many canopy variables, as is the case for PROINFORM, then LAIs is no longer the predominant variable, due to how LAI of the canopy is calculated in the model, others values such as CD, SD, and H have to be taken into consideration [88]. The greenness index NDVI reaches almost a 50% *S_Ti_* for PROSAIL. A more optimistic value is reported in [91], yet the same trend is observed in both cases: high sensitivity of LAI followed by Cab. This pattern can be observed in LAI-sensitive indices like DVI or NDVI, which are based on the comparison of a band in the red against another in the NIR [98], similar to LCC-sensitive indices. Another notable pattern is the exploiting of bands that are not influenced by Cab or water absorption, such as the DLAI or LAIDI indices, where the bands used are in the range of 970–1050 nm and 1725 nm. These kinds of indices are particularly promising for sensors that cover the SWIR range, such as EnMap [99].

Altogether, the conducted GSA exercises underline that VIs are never exclusively sensitive to a single targeted variable; all indices are affected by a greater or lesser extent by confounding variables. This is not surprising, given that canopy reflectance is the result of a complex interplay between absorbances and scattering of biochemicals and structural variables [100]. It suggests that analyzed VIs are above all greenness indices, and we should be careful with categorizing them according specific sensitivity properties. As was demonstrated here, by running RTMs the contributions of biochemical and structural variables can be quantified, for VIs as well for the full spectrum. The here presented GSA tool can as such contribute to the development of new generation indices e.g. in view of upcoming imaging spectrometer missions, not only by relying on a few bands but rather by making use of spectral shapes (e.g., integrals, derivatives) at sensitive regions. Also, although in this work sun-target-sensor geometry was not considered because only nadir sensors were analyzed, follow-up studies should also take into account the effects of geometry, since reflectance anisotropy also play a role [4,101,102]. Also further upscaling is possible. A related study explored the possibility of coupling canopy RTM with atmospheric RTM [27] proving the additional influence of atmospheric factors. However, this type of analysis requires a large amount of time due to the computational cost of current atmospheric RTM, so alternative solutions have been explored, as discussed below.

### Limitations and Opportunities in RTM-GSA Studies

Models are always a simplification of reality. A well-known limitation of the SAIL model is its absence of realism in canopy structure, as the leaf elements are organized in a turbid medium. This limitation has been partly resolved with INFORM, where explicit structural forest canopy variables have been introduced. Yet, in INFORM tree crowns are based on the SAIL principles. For more realistic realistic canopy configurations, one needs to move towards ray tracing RTMs (e.g. FLIGHT, DART) [103,104], Monte Carlo ray tracing RTM [105,106] or 3D RTM [107], where all canopy elements are explicitly defined (see comparison of these models by [108]). However, as was demonstrated in Figure 1, GSA requires many simulations to achieve stable results, typically 2000 samples per variable. When many variables are involved this easily leads to several ten thousand simulations, which may take too long computational time in case of ray tracing where each simulation implies the rendering of a scene wavelength per wavelength. Parallel processing would be a good option to reduce the computation time, e.g., only for the wavelengths that take part in the calculation of a VI. An alternative approach to bypass the burdensome processing time is to approximate the original RTM by means of the surrogate model through emulation [109]. Emulators are statistical constructs that enable to approximate the outputs of the original RTMs, but this at low computation cost so that a large number of simulations can be produced in a short time [110,111]. Recent experimental studies demonstrated accurate performances in the emulation of the leaf, canopy and atmosphere RTMs including PROSAIL, SCOPE and MODTRAN [25,112–114]. The GSA toolbox has recently been updated with an option that apart from original RTMs also their emulated counterparts can be used for GSA calculation. It would, therefore, be of interest to explore in follow-up studies: (1) the consistency of GSA results from emulation-based VIs as opposed to original RTM VIs, and if consistent, (2) apply GSA to VIs for complex, heterogeneous canopies as emulated from original ray tracing models or RTM 3D.

Related to this approach, given that RTM run-time time should no longer be a drawback with parallel computing or emulation, opportunities have opened up to evaluate spectral indices beyond the common vegetation properties using advanced RTMs. One example involves the emergence of indices for the exploitation and interpretation of sun-induced chlorophyll fluorescence spectral data [115,116], which can be calculated from the RTM SCOPE [117]. Another example involves the calculation of water indices [118], e.g., as calculated from the water RTM Hydrolight [119].

As a final remark, regardless of the realism capability of the RTM under study, it is well understood that models, at best, can only approximate reality. Hence, the observed findings on the indices’ behavior serve merely as guidelines on how indices would behave when calculated from spectral measurements over real vegetated surfaced. In this respect, for specific applications and when field data is available, GSA studies can be customized by setting the variable boundaries according to the area of study. The input variables of interest and their boundaries can easily be defined in the GSA toolbox.

## 7 Conclusions

VIs are widely used in optical remote sensing as fast indicators of biophysical variables of a vegetated surface. Yet their robustness as an indicator of a variable of interest is not fully clarified, as many factors drive variability in reflectance of vegetated surfaces. To be able to quantify the robustness of VIs, we demand a systematic and rigorous evaluation, thereby introducing a widest possible variety of biochemical and structural variability in canopy scenarios. Such kinds of exercises can be achieved with leaf and canopy RTMs, whereby multiple input variables can simulate a diversity of vegetation scenarios. In order to evaluate multiple VIs in an efficient way, we have extended ARTMO’s GSA toolbox so that the sensitivity and thus the robustness of multiple VIs can be effectively analyzed. The toolbox can be freely downloaded at https://artmotoolbox.com/. To demonstrate its utility, we analyzed the indices that were designed to respond sensitive towards LCC, LWC, and LAI and are applicable to ongoing operational satellite Earth observers (Landsat 8, MODIS, Sentinel-2, and Sentinel-3). GSA total sensitivity results suggest the following key findings: (1) none of the indices responded exclusively sensitive towards a targeted variable; (2) LCC-sensitive indices behave generally most robust towards leaf chlorophyll content with *S_Ti_* up to 84%; (3) LWC-sensitive indices responded the least robust towards LWC; they are strongly influenced by structural variables such as LAI; and, (4) LAI-sensitive indices, in turn, respond sensitively to a mixture of structural variables but also to leaf chlorophyll content. When moving from broadband indices to narrowband indices, e.g., as can be derived from the imaging spectrometer EnMAP, substantially more indices can be analyzed, yet the analyzed indices revealed the same patterns. Summarizing, all the analyzed indices are to some extent affected by variability in leaf and canopy variables, meaning that VIs are never exclusively sensitive to a single vegetation variable. This suggests that utmost care is required when applying these indices as vegetation properties indicators, especially when using them for mapping applications over heterogeneous canopies.

## Figures and Tables

**Table 1:** Main characteristics of analyzed sensors. ⋆SR: spatial resolution.

	Landsat 8	MODIS	Sentinel-2	Sentinel-3	EnMap
**Full Name**		Moderate-resolution Imaging Spectroradiometer			Environmental Mapping and Analysis Program
**Bands**	11	36	13	OLCI: 21/SLSTR: 9	230
**Spectrum [nm]**	435–12,510	405–14,385	433–2280	OLCI: 400–1020 SLSTR: 554–12,022	420–2450
**⋆SR [m]**	15–100	250–1000	10–60	300–1200	30–30
**Inclination**	98	98.2	98.6	98.65	97.98
**Orbit Height [km]**	708	705	797	814.5	653
**Orbit Type Platform**	Sun-synchronous	Sun-synchronous circular Terra/Aqua	sun-synchronous Sentinel-2	polar, sun-synchronous Sentinel-3	Sun-synchronous
**Operator**	NASA/USGS	NASA	ESA	EUMETSAT	DLR/GFZ
**Launch Date**	20-02-2011	18-12-1999	23-06-2015	16-02-2016	2020

**Table 2:** LCC-sensitive indices organized per sensor. Indices are selected according to [40].

Index	Abbreviation	Formula	References
**LandSat 8, MODIS, Sentinel 2 and Sentinel 3**			
Chlorophyll vegetation index	CVI	$NIR\frac{RED}{GREEN^{2}}$	[41]
Chlorophyll index green	CIgreen	$\frac{NIR}{\text{GREEN}}-1$	[42–44]
Green leaf index	GLI	$\frac{2\cdot GREEN-RED-BLUE}{2\cdot GREEN+RED+BLUE}$	[42,45]
Green NDVI	GNDVI	$\frac{NIR\;-\;GREEN}{NIR\;+\;GREEN}$	[7,46]
Green Ratio Vegetation Index	GRVI	$\frac{NIR}{GREEN}$	[47]
Simple Ratio 550/800	SR:550/800	$\frac{\rho_{550}}{\rho_{800}}$	[7]
**Sentinel 2 and Sentinel 3**			
Chlorophyll IndexRedEdge	CIrededge	$\frac{NIR}{\text{rededge}}-1$	[42–44]

**Table 3:** LWC-sensitive indices organized per sensor. Indices are selected according to [40].

Index	Abbreviation	Formula	References
**LandSat 8, MODIS, Sentinel 2 and Sentinel 3**			
Modification of normalized difference water index	MNDWI	$\frac{\text{GREEN}-\rho_{1500-1700}}{\text{GREEN}+\rho_{1500-1700}}$	[48]
Moisture stress index	MSI	$\frac{\rho_{1600}}{\rho_{820}}$	[49,50]
Shortwave infrared water stress index	SIWSI	$\frac{\rho_{800}-\rho_{1640}}{\rho_{800}+\rho_{1640}}$	[51]
**MODIS, Sentinel 2 and Sentinel 3**			
Normalized difference water index	NDWI	$\frac{\rho_{860}-\rho_{1240}}{\rho_{860}+\rho_{1240}}$	[51–53]
**Sentinel 3**			
Leaf water vegetation index-2	LWVI2	$\frac{\rho_{1094}-\rho_{1205}}{\rho_{1094}+\rho_{1205}}$	[54]

**Table 4:** LAI-sensitive indices organized per sensor. Indices are selected according to [40].

Index	Abbreviation	Formula	References
**LandSat 8, MODIS, Sentinel 2 and Sentinel 3**			
Corrected transformed vegetation index	CTVI	$\frac{NDVI+0.5}{NDVI+0.5}\cdot \sqrt{NDVI+0.5}$	[55]
Difference vegetation index	DVI	$\frac{NIR}{RED}$	[7,56,57]
Enhanced vegetation index	EVI	$2.5\frac{\text{NIR-RED}}{(\text{NIR}+6\text{red}-7.5\text{BLUE})+1}$	[42,58,59]
Modified single ratio	MSR	$\frac{\rho_{800}-\rho_{445}}{\rho_{680}-\rho_{445}}$	[60,61]
Normalized difference vegetation index	NDVI	$\frac{NIR-RED}{NIR+RED}$	[62,63]
Specific leaf area vegetation index	SLAVI	$\frac{NIR}{RED+SWIR}$	[64]
Wide dynamic range vegetation index	WDRVI	$\frac{0.1\cdot NIR-RED}{0.1\cdot NIR+RED}$	[44,65]

**Table 5:** Indices organized per application and selected bands for EnMAP [40]—BLUE: 449.25 nm, GREEN: 527.25 nm, RED: 670.25 nm, NIR: 1085 nm, RedEdge: 709.25 nm, SWIR: 2195 nm.

Index	Abbreviation	Formula	References
**LCC**			
Chlorophyll vegetation index	CVI	$NIR\frac{RED}{GREEN^{2}}$	[41]
Chlorophyll index green	CIgreen	$\frac{NIR}{GREEN}-1$	[42–44]
Green leaf index	GLI	$\frac{2\cdot GREEN-RED-BLUE}{2\cdot GREEN+RED+BLUE}$	[42,45]
Green NDVI	GNDVI	$\frac{NIR-GREEN}{\text{NIR}+\text{GREEN}}$	[7,46]
Green ratio vegetation index	GRVI	$\frac{\text{NIR}}{\text{GREEN}}$	[47]
Simple Ratio 550/800	SR:550/800	$\frac{\rho_{550}}{\rho_{800}}$	[7]
Chlorophyll Index Red-Edge	CIrededge	$\frac{\text{NIR}}{\text{Rededge}}-1$	[44]
Chlorophyll Red-Edge	Chlrededge	$(\frac{\text{Rededge}}{Red}{)}^{-1}$	[74]
Double difference index	DD	$\left(\rho_{749}-\rho_{720}\right)-\left(\rho_{701}-\rho_{672}\right)$	[7,60]
Double peak index	DPI	$\frac{\rho_{698}+\rho_{710}}{{\left(\rho_{697}\right)}^{2}}$	[53,60]
Green ratio vegetation index hyper	GRVIHyper	$\frac{\rho_{560}}{\rho_{658}}$	[75]
Transformed chlorophyll absorption ratio	TCARI	$3((\rho_{700}-\rho_{670})-0.2(\rho_{700}-\rho_{550})(\frac{\rho_{700}}{\rho_{670}}))$	[42,60]
Triangular chlorophyll index	TCI	$1.2\left(\rho_{700}-\rho_{550}\right)-1.5\left(\rho_{670}-\rho_{550}\right)\sqrt{\frac{\rho_{700}}{\rho_{670}}}$	[42]
**LWC**			
Modification of normalized difference water index	MNDWI	$\frac{GREEN-\rho_{1605}}{GREEN+\rho_{1605}}$	[48]
Moisture stress index	MSI	$\frac{\rho_{1600}}{\rho_{820}}$	[49,50]
Shortwave infrared water stress index	SIWSI	$\frac{\rho_{800}-\rho_{1640}}{\rho_{800}+\rho_{1640}}$	[51]
Normalized difference water index	NDWI	$\frac{\rho_{860}-\rho_{1240}}{\rho_{860}+\rho_{1240}}$	[51–53]
Leaf water vegetation index-2	LWVI-2	$\frac{\rho_{1094}-\rho_{1205}}{\rho_{1094}+\rho_{1205}}$	[54]
Disease water stress index	DSWI	$\frac{\rho_{802}+\rho_{547}}{\rho_{1657}+\rho_{682}}$	[54]
Disease water stress index-1	DSWI-1	$\frac{\rho_{800}}{\rho_{1660}}$	[76]
Leaf water vegetation index-1	LWVI-1	$\frac{\rho_{1094}-\rho_{983}}{\rho_{1094}+\rho_{983}}$	[54]
Normalized difference infrared index	NDII	$\frac{\rho_{819}-\rho_{1649}}{\rho_{819}+\rho_{1649}}$	[77]
Water band index	WBI	$\frac{\rho_{970}}{\rho_{902}}$	[78]
Water band index-4	WBI4	$\frac{\rho_{895}}{\rho_{972}}$	[79]
Water content	WC	$\frac{\rho_{1193}}{\rho_{1126}}$	[80]
Water Index	WI	$\frac{\rho_{900}}{\rho_{970}}$	[81]
Three-band ratio 1200	Ratio1200	$2\frac{\rho_{1205}}{\rho_{1095}+\rho_{1275}}$5	[82]
**LAI**			
Corrected Transformed Vegetation Index	CTVI	$\frac{NDVI+0.5}{NDVI+0.5}\cdot \sqrt{NDVI+0.5}$	[55]
Difference Vegetation Index	DVI	$\frac{NIR}{RED}$	[7,56,57]
Enhanced Vegetation Index	EVI	$2.5\frac{NIR-RED}{(NIR+6RED-7.5BLUE)+1}$	[42,58,59]
Modified single ratio	MSR	$\frac{\rho_{800}-\rho_{445}}{\rho_{680}-\rho_{445}}$	[60,61]
Normalized difference vegetation index	NDVI	$\frac{NIR-RED}{\text{NIR}+\text{RED}}$	[62,63]
Specific Leaf Area Vegetation Index	SLAVI	$\frac{\text{NIR}}{\text{RED}+\text{SWIR}}$	[64]
Wide Dynamic Range Vegetation Index	WDRVI	$\frac{0.1\cdot NIR-RED}{0.1\cdot NIR+RED}$	[44,65]
Difference 1725/970 Difference LAI	DLAI	*ρ*_1725_ – *ρ*_970_	[8]
Simple Ratio 1250/1050 LAI determining index	LAIDI	$\frac{\rho_{1250}}{\rho_{1050}}$	[83]

**Table 6:** Parameters considered in the data simulations. The observer zenith angle is kept at nadir (0°). The fraction of diffuse radiation and hot spot parameter are kept at their default value (SAIL parameters).

Input	Description	Unit	Min	Max
**Leaf: PROSPECT4**				
N	Leaf structural parameter	[-]	1	2.6
Cab	Chlorophyll a+b content	[μg/cm^2^]	0	80
Cw	Equivalent water thickness	[g/cm^2^] or [cm]	0.001	0.08
Cm	Dry matter content	[g/cm^2^]	0.001	0.02
**Canopy: SAIL and INFORM**				
LAD	Leaf angle distribution	[°]	0	90
SZA *θ_S_*	Solar Zenith Angle	[°]	0	60
*ρ_S_*	Soil Coefficient	[-]	0	1
**Canopy: only SAIL**				
LAI	Total leaf area index	[m^2^/m^2^]	0	10
**Canopy: only INFORM**				
LAIs	Single tree leaf area index	[m^2^/m^2^]	0	10
LAIu	Leaf area index of understory	[m^2^/m^2^]	0	5
SD	Stem density	[1/ha]	0.5	1500
H	Tree height	m	0.5	30
CD	Crown diameter	m	0.1	10
